# Acute Proximal Myopathy in a Young Male—A Case of Infectious Myositis

**DOI:** 10.3390/medicina55010019

**Published:** 2019-01-17

**Authors:** Rashmi Dhital, Sijan Basnet, Dilli Ram Poudel

**Affiliations:** 1 Reading Hospital, Tower Health System, West Reading, PA 19611, USA; sijanbasnet@gmail.com; 2 University of Pennsylvania, Philadelphia, PA 19104, USA; dr.dillirampoudel@gmail.com

**Keywords:** infectious myositis, myopathy

## Abstract

*Background and objectives*: Acute proximal muscle weakness has a broad differential. Infectious myositis is difficult to differentiate clinically from inflammatory myopathy, often causing a delayed diagnosis. Infectious myositis should be thought of as a differential for proximal muscle pain and weakness in the right context. *Case Presentation*: A 40-year-old male with diabetes presented with exquisite pain and weakness of proximal extremities. He denied trauma, recent travel, new medications, or substance use. He denied prior rheumatologic, thyroid, or musculoskeletal disorders. The urine culture revealed staphylococcal infection with negative blood cultures. Rheumatologic and endocrine workups were negative. Random muscle biopsy was negative for inflammatory infiltrate. MRI of thighs and arms showed innumerable foci of nodular and ring enhancement in the proximal muscle groups. The patient noted improvement after about 10 days of antibiotics with complete resolution at 2 months. *Discussion and Conclusion*: Bacterial myositis is most often due to *Staphylococcus aureus* (70%) and affects a single muscle. Multifocal abscesses are rare and strongly suggest transient bacteremia. Our patient most likely had transient initiating staphylococcal bacteremia leading to diffuse myositis and hematogenous urinary tract infection (UTI). A delay in treatment can be life-threatening.

## 1. Introduction

Acute symmetrical proximal muscle weakness has a broad differential that includes inflammatory, infectious, metabolic or drug-induced myositis, endocrinopathies, connective tissues diseases, polyneuropathies, and neuromuscular junction disorders [1]. Infectious myositis is difficult to differentiate clinically from inflammatory myopathy, often causing a delayed diagnosis. Prompt administration of antibiotics can result in a complete resolution of infectious myositis, whereas delay in treatment may result in life-threatening complications including sepsis and septic shock [2].

## 2. Case Presentation

A 40-year-old male with a medical history significant for diabetes mellitus and hypertension presented to the emergency department (ED) with complaints of progressively worsening proximal muscle weakness of bilateral lower and upper extremities of 4 days duration. The pain and weakness was reported to be worse in lower extremities. He denied fever, chills, recent trauma, or strenuous activities. He denied rash, photosensitivity, back pain, abdominal pain, dysphagia, diarrhea, dysuria, or incontinence. History was negative for sick contacts or recent travel. He denied recent illnesses, new medications (such as statins or exogenous steroids), alcohol intake, or recreational drug use. He denied prior rheumatologic or thyroid disorders and a personal or family history of musculoskeletal disorders.

At presentation, he was noted to have extreme difficulty standing from the wheelchair. He was initially afebrile but subsequently developed a low-grade fever (maximum temperature of 101.3 °F). Exquisite tenderness on palpation of the proximal muscle groups of upper and lower extremities was present without atrophy. Muscle strength at initial examination was noted as 4/5 on bilateral shoulder abductors, elbow flexors, and extensors; 5/5 on bilateral wrist flexors and extensors; 2/5 on bilateral hip flexors; 3/5 on bilateral quadriceps; and 5/5 on bilateral plantar flexors and extensors. No heliotrope rashes were noted. Neurological examination was non-focal.

Lab tests were notable for slight leukocytosis (white cell count (WBC) of 11 × 103/μL (ref, 4.8–10.8 × 103/μL)) with increased bands. Blood glucose was elevated at 394 (ref, 70–99) mg/dL with hemoglobin A1c of 11%. Urinalysis was consistent with urinary tract infection (UTI) with many bacteria, 26 WBCs, and positive nitrite, with a normal renal ultrasound. UTI at admission and was initially empirically treated and later changed to Cefazolin after urine culture grew methicillin-sensitive *Staphylococcus aureus* (MSSA). Blood culture was not obtained at the time of presentation; however, was sent after positive urine culture, after about 2 days of antibiotic therapy, and was negative. Alongside, further workups were done to determine the etiology of myopathy, including multiple metabolic, infectious, and rheumatologic workups.

- Electrolytes (Na, K, Ca, P, and Mg) within normal limits.
- Elevated inflammatory markers- C-reactive protein (CRP) of 24.33, ref: <1 mg/dL; Erythrocyte sedimentation rate (ESR) of 40, ref: 0–15 mm/h.
- Mildly elevated creatinine kinase (CK) of 273 (ref, 30–223) IU/L; normal transaminases; aldolase 6.1 (ref, 1.5–8.1) U/L; and lactate dehydrogenase of 223 (ref, 140–271) IU/L.
- Unremarkable vitamin B12, folate, and thyroid stimulating hormone levels.
- Negative urinary drug screen.
- Negative HIV screen.
- Negative serology for Lyme disease IgM and IgG; Parvovirus B19 antibody IgM; and Respiratory syncitial virus and Influenza A and B.
- No monoclonal proteins on protein electrophoresis.
- Negative serological tests for inflammatory myopathy or associated connective tissue diseases.
  ▪ Anti-nuclear antibodies (ANA)-negative 1:40; Anti-ds DNA-negative.
  ▪ Antibodies against extractable nuclear antigens-negative anti-Ro and anti-La.
  ▪ “Myositis specific” autoantibodies-e.g., Jo^−1^ IgG antibody of <0.3 (ref ≤ 6.9) U/mL; signal recognition particle (SRP) and Mi-2 autoantibodies not detected; and cytoplasmic 5′-nucleotidase 1A (cN1A) Ab < 20 (ref < 20) units.
- Negative serological tests for vasculitis.
  ▪ Anti-neutrophil cytoplasmic antibody (ANCA) titers-MPO IgG Ab < 0.3 (ref ≤ 3.4 U/mL) and PR3 IgG Ab < 0.7 (ref ≤ 1.9 U/mL).
  ▪ Negative Hepatitis B and C serology.

After all the initial workups and serology were negative, imaging was considered.

**Figure 1:**
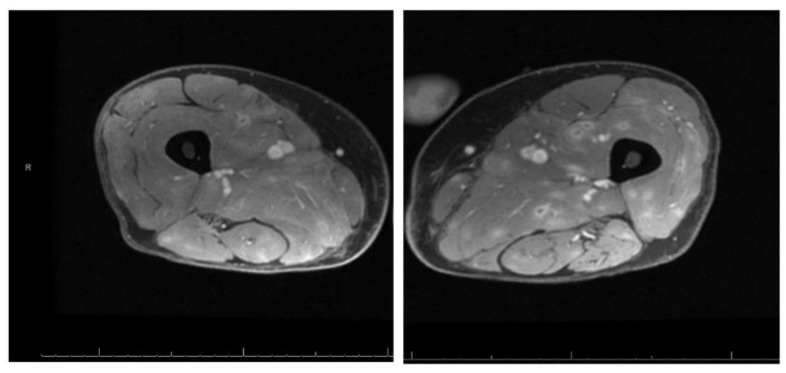
MRI of bilateral thighs with diffuse edema and multiple nodular and ring enhancing lesions.

- On further evaluation with MRI of the extremities and lumbar spine,
  ▪ MRI of the bilateral thighs showed diffuse muscle edema with innumerable foci of nodular and ring enhancement-concerning for diffuse infectious myositis (Figure 1).
  ▪ MRI lumbar spine with no evidence of diskitis or osteomyelitis but showed multiple small foci of intramuscular enhancement, including the iliopsoas, gluteal muscles, and posterior paravertebral muscles and possible left perinephric inflammation.
- Similarly, MRI of the upper extremities showed muscle edema within supraspinatus, infraspinatus, subscapularis, deltoid, and triceps and to a lesser extent, the biceps muscles with multiple small foci of nodular and ring-like enhancement in the visualized muscles of the upper arm and proximal forearm.
- CT-guided aspiration of the left thigh was attempted only after about 4 days of treatment with antibiotics (no focal fluid collection was noted on CT and random muscle biopsy was obtained). No growth on cultures, including acid fast bacilli, anaerobic and fungal cultures, and no significant inflammatory infiltrate was reported.
- During the hospital stay, his CK had improved to 81 IU/L on repeat lab tests at 4 days.

**Figure 2:**
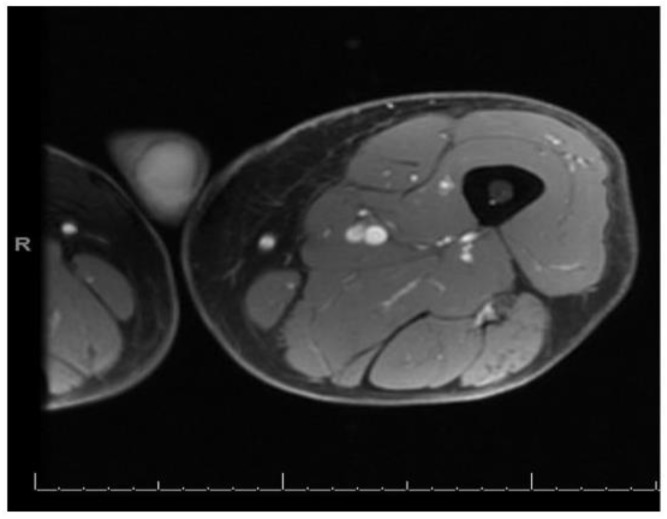
MRI of left thigh with resolution of diffuse edema and previous foci of multiple nodular and ring enhancing lesions.

After almost about 10 days of antibiotics, the patient started to have a gradual improvement in pain and muscle weakness. He was discharged in a stable condition at day 14 to continue 2 more weeks of oral antibiotics and with recommendation for outpatient follow up. After 2 months of initial presentation, patient was seen at his family physician’s office at which time he had complete resolution of the weakness and diffuse muscle pain. However, he complained of one tender lump at the distal and medial aspect of left thigh and there was no sign of infection. Outpatient MRI of the left thigh showed resolution of previous foci of abnormal signal within the thigh musculature (Figure 2); however, a new 2.5 cm peripherally enhancing lesion within the vastus medialis muscle was seen, with possible differentials of hematoma, abscess, and myonecrosis. An ultrasound guided aspiration of the mass was attempted; the ultrasound, however, did not show a loculated fluid collection, and core biopsies were obtained from the distal medial left thigh from a location with altered architecture as noted in MRI. The tissue specimen was reported as skeletal muscle with endomysial and perimysial chronic inflammation and atrophy. Microscopic evaluation showed no polymorphonuclear leukocytes and no growth on cultures. An immune-histochemical stain was positive for leukocyte common antigen (LCA) confirming a marked lymphocytic component, with plans for further special histochemical studies.

### Ethical Statement

Informed consent was obtained from the patient for submission of the case.

## 3. Discussion

A careful history and physical examination helps distinguish functional limitation, motor impairment due to joint pain, and true muscle weakness from each other. Muscle tenderness is usually absent with true muscle weakness, except in certain instances including infectious, drug-induced, thyroid, and inherited metabolic myopathies [3]. Rim-enhancing lesions in muscle may be cysts, post-operative seromas, hematomas, necrotic tumor masses, or abscesses [4].

Bacterial infection of the muscle, or pyomyositis, is most often due to *Staphylococcus aureus* (70%), and usually affects a single muscle, and is more predominant among young males [2,5]. Diabetes mellitus has been established as an important risk factor [5]. Multifocal abscesses are relatively rare (about 15%) and strongly suggest an initiating staphylococcal bacteremia; however, blood cultures positive in <5% cases suggest that bacteremia occurs early and is transient [6]. The infecting organism is frequently not isolated from cultures of purulent materials as well [5]. In our patient, MSSA on urine culture could be indicative of blood stream infection, resulting in diffuse muscle involvement; however, blood culture was not obtained at admission.

Infectious myositis is difficult to distinguish clinically from inflammatory myositis, sometimes causing a delay in diagnosis. With no signs of rheumatic disease and especially in the setting of an acute infection, infectious myositis should be a differential for proximal muscle weakness. MRI is the modality of choice to accurately assess the extent of muscle involvement. In our patient, the intramuscular collection could not be visualized and sampled by CT-guided biopsy.

Our patient most likely had initiating transient MSSA bacteremia leading to both diffuse myositis and hematogenous UTI. Also, following treatment with antibiotics, the numerous ring enhancing lesions had resolved, further supporting the infectious etiology of the problem. While early recognition and treatment can resolve the infection, delayed diagnosis may result in severe complications.

## 4. Conclusions

Acute symmetrical proximal muscle weakness has varied etiologies. Infectious myositis is difficult to differentiate clinically from inflammatory myopathy, often causing a delayed diagnosis, leading to multiple downstream investigations and a delay in diagnosis.
